# 6-Methyl-2-phenyl-4,5-dihydro­pyridazin-3(2*H*)-one

**DOI:** 10.1107/S1600536811021970

**Published:** 2011-06-18

**Authors:** Hanan Sekkak, El Mostapha Rakib, Hafid Zouihri

**Affiliations:** a Laboratoire de Chimie Organique et Analytique, Université Sultan Moulay Slimane, Faculté des Sciences et Techniques, Béni-Mellal, BP 523, Morocco; b Laboratoires de Diffraction des Rayons X, Centre Nationale pour la Recherche Scientifique et Technique, Rabat, Morocco

## Abstract

In the title mol­ecule, C_11_H_12_N_2_O, the pyridazine ring has a skew-boat conformation. The dihedral angle between the phenyl ring [r.m.s deviation = 0.0039 (15) Å] and the best mean-plane of the pyridazine ring [r.m.s deviations = 0.2629 (15) Å] is 53.27 (10)°. In the crystal, mol­ecules are linked by C—H⋯O hydrogen bonds and C—H⋯π inter­actions involving the methyl group and the phenyl ring of a symmetry-related mol­ecule.

## Related literature

For the similar structure, 2-(4-meth­oxy­phen­yl)-6-(trifluoro­meth­yl)-4,5-dihydro­pyridazin-3(2*H*)-one, see: Wan *et al.* (2009 ▶). For conformation analysis of six-membered rings, see: Cremer & Pople (1975 ▶).
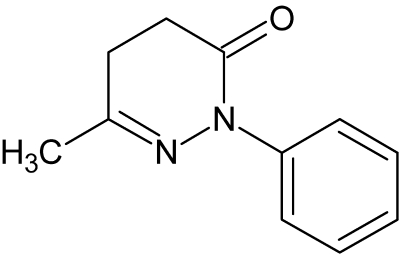


## Experimental

### 

#### Crystal data

C_11_H_12_N_2_O
*M* *_r_* = 188.23
Monoclinic, 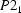

*a* = 6.4151 (2) Å
*b* = 7.9010 (2) Å
*c* = 10.1888 (3) Å
β = 106.607 (1)°
*V* = 494.89 (2) Å^3^
*Z* = 2
Mo *K*α radiation
μ = 0.08 mm^−1^
*T* = 296 K
0.24 × 0.15 × 0.12 mm

#### Data collection

Bruker APEXII CCD detector diffractometer
7678 measured reflections
1220 independent reflections
1154 reflections with *I* > 2σ(*I*)
*R*_int_ = 0.031

#### Refinement

*R*[*F* ^2^ > 2σ(*F* ^2^)] = 0.032
*wR*(*F* ^2^) = 0.094
*S* = 1.06
1220 reflections
129 parameters
1 restraint
H-atom parameters constrained
Δρ_max_ = 0.15 e Å^−3^
Δρ_min_ = −0.13 e Å^−3^

### 

Data collection: *APEX2* (Bruker, 2005 ▶); cell refinement: *SAINT* (Bruker, 2005 ▶); data reduction: *SAINT*; program(s) used to solve structure: *SHELXS97* (Sheldrick, 2008 ▶); program(s) used to refine structure: *SHELXL97* (Sheldrick, 2008 ▶); molecular graphics: *PLATON* (Spek, 2009 ▶); software used to prepare material for publication: *publCIF* (Westrip, 2010 ▶).

## Supplementary Material

Crystal structure: contains datablock(s) I, Global. DOI: 10.1107/S1600536811021970/su2275sup1.cif

Structure factors: contains datablock(s) I. DOI: 10.1107/S1600536811021970/su2275Isup2.hkl

Supplementary material file. DOI: 10.1107/S1600536811021970/su2275Isup3.cml

Additional supplementary materials:  crystallographic information; 3D view; checkCIF report

## Figures and Tables

**Table 1:** Hydrogen-bond geometry (Å, °) *Cg*1 is the centroid of the C1–C6 ring.

*D*—H⋯*A*	*D*—H	H⋯*A*	*D*⋯*A*	*D*—H⋯*A*
C3—H3⋯O1^i^	0.93	2.54	3.371 (3)	149
C5—H5⋯O1^ii^	0.93	2.50	3.346 (2)	152
C8—H8*B*⋯O1^iii^	0.97	2.55	3.474 (3)	159
C11—H11*B*⋯*Cg*1^iv^	0.96	2.89	3.812 (3)	161

Symmetry codes: (i) 
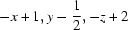
; (ii) 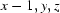; (iii) 
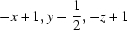
; (iv) 
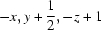
.

**Fig. 1.**
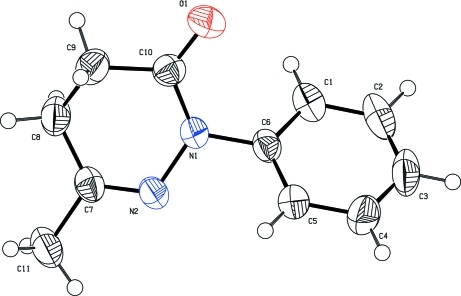
A view of the molecular structure of the title molecule, showing the atom-labeling scheme. Displacement ellipsoids are drawn at the 50% probability level.

**Fig. 2.**
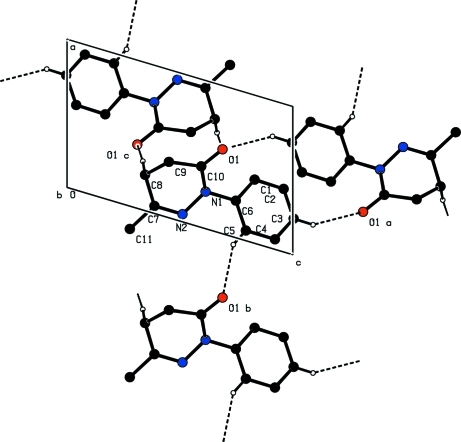
A view along the b axis of the crystal packing of the title compound, showing the chain formed by C—H···O interactions (dashed lines; see Table 1 for details). H-atoms not involved in hydrogen bonding have been omitted for clarity.

## supplementary crystallographic information

### Comment

The molecular structure of the title molecule is illustrated in Fig. 1. The bond distances and angles are simliar to those reported for 2-(4-Methoxyphenyl)-6-(trifluoromethyl)-4,5-dihydropyridazin-3(2*H*)-one (Wan *et al.*, 2009). The pyridazine ring has a skew-boat conformation; Puckering Amplitude (Q) = 0.428 (2) Å, θ = 69.9 (2)°, φ = 207.6 (3) ° (Cremer and Pople, 1975). The dihedral angle between the phenyl ring and the best mean-plane of the pyridazine ring (r.m.s deviations: 0.2629 (15) Å) is 53.27 (10)°.

In the crystal molecules are linked *via* non-classical C—H···O hydrogen bonds (Table 1), forming a two-dimensional network (Fig. 2). Molecules are also linked by C—H···π interactions involving a methyl H-atom and the phenyl ring of a symmetry related molecule (Table 1).

### Experimental

A mixture of phenylhydrazine (2.7 ml, 27 mmol) and levulinic acid (2.6 ml, 25 mmol) in 60 ml of ethanol were refluxed for 4 h. After cooling the reaction mixture was poured onto ice. The solid obtained was filtered off and recrystallized from methanol to give the title compound as colourless crystals: Yield 3.9 g (85%); Mp: 367–369 K. Spectroscopic data for the title compound is given in the archived CIF.

### Refinement

In the final cycles of refinement, in the absence of significant anomalous scattering effects, 906 Friedel pairs were merged and Δf " set to zero. H-atoms were positioned geometrically, with C–H = 0.93 Å for CH(aromatic), 0.97 Å for CH_2_ and 0.96 Å for CH_3_ H-atoms. They were constrained to ride on their parent atoms, with *U*_iso_(H) = k × *U*_eq_(C), where k = 1.5 for CH_3_ H-atoms and k = 1.2 for all other H-atoms.

### Crystal data

**Table:**

C_11_H_12_N_2_O	*F*(000) = 200
*M**_r_* = 188.23	*D*_x_ = 1.263 Mg m^−^^3^
Monoclinic, *P*2_1_	Melting point: 397 K
Hall symbol: P 2yb	Mo *K*α radiation, λ = 0.71073 Å
*a* = 6.4151 (2) Å	Cell parameters from 256 reflections
*b* = 7.9010 (2) Å	θ = 2.4–26.5°
*c* = 10.1888 (3) Å	µ = 0.08 mm^−^^1^
β = 106.607 (1)°	*T* = 296 K
*V* = 494.89 (2) Å^3^	Prism, colourless
*Z* = 2	0.24 × 0.15 × 0.12 mm

### Data collection

**Table:**

Bruker APEXII CCD detector diffractometer	1154 reflections with *I* > 2σ(*I*)
Radiation source: fine-focus sealed tube	*R*_int_ = 0.031
graphite	θ_max_ = 27.5°, θ_min_ = 2.1°
ω and φ scans	*h* = −8→7
7678 measured reflections	*k* = −8→10
1220 independent reflections	*l* = −12→13

### Refinement

**Table:**

Refinement on *F*^2^	Secondary atom site location: difference Fourier map
Least-squares matrix: full	Hydrogen site location: inferred from neighbouring sites
*R*[*F*^2^ > 2σ(*F*^2^)] = 0.032	H-atom parameters constrained
*wR*(*F*^2^) = 0.094	*w* = 1/[σ^2^(*F*_o_^2^) + (0.0582*P*)^2^ + 0.0443*P*] where *P* = (*F*_o_^2^ + 2*F*_c_^2^)/3
*S* = 1.06	(Δ/σ)_max_ < 0.001
1220 reflections	Δρ_max_ = 0.15 e Å^−^^3^
129 parameters	Δρ_min_ = −0.13 e Å^−^^3^
1 restraint	Extinction correction: *SHELXL*, Fc^*^=kFc[1+0.001xFc^2^λ^3^/sin(2θ)]^-1/4^
Primary atom site location: structure-invariant direct methods	Extinction coefficient: 0.21 (2)

### Special details

**Table:**

Experimental. Spectroscopic data for the title compound: ^1^*H*-NMR (CDCl_3_): δ 2.14 (s, 3H, CH3), 2.54–2.65 (m, 4H, –CH2—CH2-), 7.20–7.31 (m, 2H, H—Ar), 7.36–7–40 (m, 1H, H—Ar), 7.48–7.51 (m, 2H, H—Ar); ^13^C-NMR (CDCl_3_): δ 23.2 (CH3), 26.3 (CH2), 27.7 (CH2), 125 (2CH-Ar), 126.5 (CH—Ar), 128.6 (2CH-Ar), 141.1, 154 (2 C), 165 (C═O).
Geometry. All e.s.d.'s (except the e.s.d. in the dihedral angle between two l.s. planes) are estimated using the full covariance matrix. The cell e.s.d.'s are taken into account individually in the estimation of e.s.d.'s in distances, angles and torsion angles; correlations between e.s.d.'s in cell parameters are only used when they are defined by crystal symmetry. An approximate (isotropic) treatment of cell e.s.d.'s is used for estimating e.s.d.'s involving l.s. planes.
Refinement. Refinement of *F*^2^ against ALL reflections. The weighted *R*-factor *wR* and goodness of fit *S* are based on *F*^2^, conventional *R*-factors *R* are based on *F*, with *F* set to zero for negative *F*^2^. The threshold expression of *F*^2^ > σ(*F*^2^) is used only for calculating *R*-factors(gt) *etc*. and is not relevant to the choice of reflections for refinement. *R*-factors based on *F*^2^ are statistically about twice as large as those based on *F*, and *R*– factors based on ALL data will be even larger.

### Fractional atomic coordinates and isotropic or equivalent isotropic displacement parameters (Å^2^)

**Table:**

	*x*	*y*	*z*	*U*_iso_*/*U*_eq_	
C1	0.4300 (3)	0.0477 (3)	0.83092 (17)	0.0488 (5)	
C10	0.4122 (2)	0.2873 (2)	0.59455 (17)	0.0413 (4)	
C11	−0.1543 (3)	0.1310 (3)	0.2779 (2)	0.0598 (6)	
C2	0.4258 (4)	−0.0098 (3)	0.95947 (18)	0.0608 (6)	
C3	0.2448 (4)	0.0173 (4)	1.00372 (19)	0.0673 (7)	
C4	0.0679 (4)	0.1005 (4)	0.92101 (19)	0.0652 (6)	
C5	0.0682 (3)	0.1580 (3)	0.79218 (17)	0.0488 (4)	
C6	0.2502 (3)	0.1303 (2)	0.74779 (15)	0.0389 (4)	
C7	0.0504 (3)	0.1740 (2)	0.38723 (16)	0.0416 (4)	
C8	0.2420 (3)	0.2321 (3)	0.34560 (17)	0.0513 (4)	
C9	0.3773 (3)	0.3520 (3)	0.45107 (18)	0.0527 (5)	
H1	0.5527	0.0307	0.8012	0.059*	
H11A	−0.1265	0.0394	0.2233	0.090*	
H11B	−0.2028	0.2282	0.2207	0.090*	
H11C	−0.2646	0.0979	0.3195	0.090*	
H2	0.5454	−0.0666	1.0154	0.073*	
H3	0.2425	−0.0207	1.0897	0.081*	
H4	−0.0537	0.1187	0.9516	0.078*	
H5	−0.0522	0.2143	0.7365	0.059*	
H8A	0.1937	0.2887	0.2576	0.062*	
H8B	0.3295	0.1353	0.3359	0.062*	
H9A	0.5173	0.3679	0.4341	0.063*	
H9B	0.3053	0.4611	0.4420	0.063*	
N1	0.25017 (19)	0.18561 (19)	0.61361 (12)	0.0389 (3)	
N2	0.0518 (2)	0.1529 (2)	0.51137 (14)	0.0434 (4)	
O1	0.5696 (2)	0.3270 (2)	0.68880 (14)	0.0560 (4)	

### Atomic displacement parameters (Å^2^)

**Table:**

	*U*^11^	*U*^22^	*U*^33^	*U*^12^	*U*^13^	*U*^23^
C1	0.0539 (9)	0.0478 (11)	0.0389 (8)	0.0057 (8)	0.0038 (7)	−0.0021 (8)
C10	0.0413 (7)	0.0409 (9)	0.0420 (8)	−0.0027 (7)	0.0125 (6)	−0.0036 (7)
C11	0.0667 (12)	0.0614 (14)	0.0393 (9)	−0.0110 (11)	−0.0044 (8)	0.0058 (9)
C2	0.0803 (13)	0.0507 (11)	0.0362 (8)	0.0005 (11)	−0.0081 (8)	0.0020 (9)
C3	0.0981 (17)	0.0708 (15)	0.0300 (7)	−0.0190 (14)	0.0136 (9)	0.0020 (9)
C4	0.0708 (12)	0.0870 (17)	0.0420 (9)	−0.0164 (12)	0.0230 (9)	−0.0045 (11)
C5	0.0465 (8)	0.0620 (11)	0.0374 (8)	−0.0036 (9)	0.0112 (6)	0.0008 (8)
C6	0.0448 (8)	0.0387 (8)	0.0301 (7)	−0.0042 (7)	0.0056 (6)	−0.0015 (6)
C7	0.0499 (8)	0.0360 (8)	0.0346 (7)	−0.0007 (7)	0.0048 (6)	0.0022 (7)
C8	0.0625 (10)	0.0570 (11)	0.0352 (7)	−0.0039 (9)	0.0151 (7)	0.0019 (8)
C9	0.0579 (10)	0.0550 (11)	0.0474 (9)	−0.0115 (9)	0.0189 (8)	0.0028 (9)
N1	0.0390 (6)	0.0451 (8)	0.0306 (6)	−0.0027 (6)	0.0067 (5)	0.0004 (6)
N2	0.0416 (7)	0.0494 (9)	0.0342 (6)	−0.0062 (7)	0.0027 (5)	0.0034 (6)
O1	0.0454 (6)	0.0631 (9)	0.0533 (7)	−0.0136 (7)	0.0044 (5)	−0.0029 (7)

### Geometric parameters (Å, °)

**Table:**

C1—H1	0.9300	C5—C4	1.389 (3)
C1—C2	1.394 (3)	C6—N1	1.4351 (19)
C10—C9	1.504 (2)	C6—C5	1.385 (2)
C10—N1	1.371 (2)	C6—C1	1.384 (2)
C10—O1	1.219 (2)	C7—C11	1.498 (2)
C11—H11C	0.9600	C7—C8	1.483 (2)
C11—H11B	0.9600	C7—N2	1.273 (2)
C11—H11A	0.9600	C8—H8B	0.9700
C2—H2	0.9300	C8—H8A	0.9700
C2—C3	1.377 (4)	C9—H9B	0.9700
C3—H3	0.9300	C9—H9A	0.9700
C3—C4	1.373 (4)	C9—C8	1.507 (3)
C4—H4	0.9300	N2—N1	1.4191 (17)
C5—H5	0.9300		
			
C2—C1—H1	120.2	C6—C5—C4	119.07 (19)
C6—C1—H1	120.2	C5—C6—N1	119.50 (15)
C6—C1—C2	119.50 (19)	C1—C6—N1	119.99 (15)
N1—C10—C9	115.39 (14)	C1—C6—C5	120.50 (15)
O1—C10—C9	122.55 (16)	C8—C7—C11	118.64 (15)
O1—C10—N1	122.02 (16)	N2—C7—C11	117.69 (17)
H11B—C11—H11C	109.5	N2—C7—C8	123.63 (14)
H11A—C11—H11C	109.5	H8A—C8—H8B	108.1
C7—C11—H11C	109.5	C9—C8—H8B	109.6
H11A—C11—H11B	109.5	C7—C8—H8B	109.6
C7—C11—H11B	109.5	C9—C8—H8A	109.6
C7—C11—H11A	109.5	C7—C8—H8A	109.6
C1—C2—H2	120.0	C7—C8—C9	110.26 (15)
C3—C2—H2	120.0	H9A—C9—H9B	107.9
C3—C2—C1	120.08 (19)	C8—C9—H9B	109.2
C2—C3—H3	120.0	C10—C9—H9B	109.2
C4—C3—H3	120.0	C8—C9—H9A	109.2
C4—C3—C2	120.02 (17)	C10—C9—H9A	109.2
C5—C4—H4	119.6	C10—C9—C8	112.02 (17)
C3—C4—H4	119.6	N2—N1—C6	113.57 (12)
C3—C4—C5	120.8 (2)	C10—N1—C6	121.30 (13)
C4—C5—H5	120.5	C10—N1—N2	124.06 (13)
C6—C5—H5	120.5	C7—N2—N1	117.09 (14)

### Hydrogen-bond geometry (Å, °)

**Table:**

Cg1 is the centroid of the C1–C6 ring.

**Table:**

*D*—H···*A*	*D*—H	H···*A*	*D*···*A*	*D*—H···*A*
C3—H3···O1^i^	0.93	2.54	3.371 (3)	149
C5—H5···O1^ii^	0.93	2.50	3.346 (2)	152
C8—H8B···O1^iii^	0.97	2.55	3.474 (3)	159
C11—H11B···Cg1^iv^	0.96	2.89	3.812 (3)	161

Symmetry codes: (i) −*x*+1, *y*−1/2, −*z*+2; (ii) *x*−1, *y*, *z*; (iii) −*x*+1, *y*−1/2, −*z*+1; (iv) −*x*, *y*+1/2, −*z*+1.
